# New Conceptional Study of a Portable Highly Sensitive Photometric Raman Sensor

**DOI:** 10.3390/s22166098

**Published:** 2022-08-15

**Authors:** Steffen Manser, Sandy Kommert, Shaun Keck, Erik Spoor, Matthias Rädle

**Affiliations:** 1 Center for Mass Spectrometry and Optical Spectroscopy (CeMOS), University of Applied Science Mannheim, 68163 Mannheim, Germany; 2 Hellma GmbH & Co. KG, 79379 Müllheim, Germany

**Keywords:** process engineering, Raman effect, photometry, batch process monitoring, optical measurement, highly sensitive detector, explosive atmosphere

## Abstract

Quality control and reaction monitoring are necessary to ensure the consistency of any kind of mixing or reaction process. In this context, a novel portable high-sensitivity sensor prototype based on the Raman effect is presented in this study. The elongated probe head is designed for (but not limited to) monitoring high temperature batch processes for quality assurance. Thanks to the highly sensitive special detectors, concentration differences of up to 50 mmol/L can currently be detected, which facilitates compliance with high product quality standards. In addition, seamless reaction tracking is possible. Small individual adjustments through simple, intuitive mechanical components provide a high degree of flexibility in reaction selection by the end user. Specially developed software automates the evaluation process and gives the user visual signals about the current status and progress of the batch as well as an emergency stop if temperature limits could damage individual components. By using all the individual components developed, the problem of the limited integration times of previous spectrometric measuring instruments could be reduced. The prototype was validated using concentration measurements of various substances that occur as standard in batch processes. In addition, this article provides an outlook on the fact that Raman measurements can also be carried out successfully and reliably in explosive environments in the future with the prototype presented.

## 1. Introduction

In this paper we present a portable highly sensitive prototype that uses the Raman effect for quality assurance and process monitoring in mixing or reaction processes that are mainly discontinuous. All measurements were performed at CeMOS—Center for Mass Spectrometry and Optical Spectroscopy, an interfaculty institution of the Mannheim University of Applied Sciences, Germany.

Batch processes are discontinuous processes in which several reactants are combined in a batch reactor, and reaction conditions are created with the aid of critical parameters [1]. Raman-active molecules exhibit spectral bands specific to covalent bonds and are suitable for selective monitoring [2]. This makes it possible to measure the decrease or increase of individual educt and product concentrations and to derive qualitative statements about the reaction progress. By selecting suitable peaks specific to the reaction, an adaptable process monitoring function is created [3]. Since reactants and their corresponding spectral data can be considered prior knowledge in industrial processes, the presented sensor can be adapted to any reaction of Raman-active molecules with the aid of simple mechanical adjustments. In addition, the prototype is designed as a portable handheld device and can therefore be used alternately for several processes and parallel monitoring of several batch processes.

The advantages of using the Raman effect in batch processes compared to conventional spectroscopic methods are manifold. For example, no sample preparation is required for measurements of the Raman effect (in comparison to NIR measurements). This means reaction data can be directly collected from inside the batch volume potentially in real time, giving access to critical information in all respective states. Additionally, Raman spectroscopy and derived measuring concepts are non-destructive measurement methods. Furthermore, the measurement process is not affected by the presence of water, e.g., as a byproduct or solvent, as opposed to IR spectroscopy. Raman spectra in the range from 1600 cm^−1^ to 50 cm^−1^ are comparable to fingerprints specific to each molecule. Measurement of the Raman effect can occur in a time range of seconds. In the system presented here, the recording occurs within a few milliseconds because of the highly sensitive sensor. On the other hand, Raman spectroscopy is not suitable for all materials because vibrating molecular bonds must be present. This excludes metals or alloys. The Raman effect itself is very weak compared to other measurement methods. This circumstance can be compensated by highly sensitive and highly optimized detection techniques. It should also be mentioned that the presence of fluorophores can mask the Raman signal, since fluorescence signals are several orders of magnitude higher and are broadband. In addition, molecule excitation by a focused laser beam can potentially cause localized damage of the sample due to thermal stress [4,5,6].

The presented prototype features the use of state-of-the-art custom photon multiplier detectors (CPM) that significantly increase sensitivity. Furthermore, a non-contact long range probe is provided to counteract thermal effects on the components. In addition, mechanical setting options are used to diversify the application range of the prototype. The photo spectrometric sensor presented in this work encompasses all necessary conditions for application-specific, pre-selected Stokes shifted reaction monitoring.

## 2. Materials and Methods

Raman spectroscopy is an analytical method for the quantitative and qualitative investigation of molecular compositions. As monochromatic light interacts with the sample, most of the light is simply scattered (Rayleigh scattering); only a fraction is briefly absorbed in the form of energy and emitted again. The photons emitted by the vibrating molecular bonds are scattered in all spatial directions. A distinction is made here between two alternative effects. If the incident photon wavelengths shifts to longer wavelengths and accordingly lower frequencies, the effect is referred to as Stokes shift. If the photon wavelengths however decrease and their frequencies accordingly increase, the effect is referred to as anti-Stokes shift. The prototype was designed for detecting Stokes-shifted Raman scattering. By shifting the wavelengths or rather changing the energy levels, a characteristic spectrum of the sample is generated. The individual peaks provide information about the molecular composition of the sample. The position indicates the characteristic group of molecular compounds, and the height of the peaks indicates the quantity. The spectral coordinate corresponds to the vibrational mode of a specific covalent bond and the peak height (intensity) shows how often this bond is present. Therefore, it can be inferred how much of a molecule is present. The occurrence of the Raman effect has a very low probability. The Stokes–Raman scattering considered here occurs in only one case out of 10^6^ [7,8].

Since the Raman effect occurs relative to the excitation wavelength, no unique signal can be detected with multichromatic excitation light sources, since the individual molecular vibrations overlap at multiple excitation wavelengths. The standard wavelengths for lasers in Raman spectroscopy are 532 nm and 785 nm. Since signal intensity is proportional to the wavelength with the fourth power, excitation wavelength has great leverage as a technical parameter. At lower wavelengths, the Raman signal is more pronounced than at higher wavelengths. However, with fluorescent materials, the fluorescence is also more pronounced, risking greater superposition of the Raman signal. Here, it was necessary to find a compromise that would be optimal for the application [9].

**Figure 1:**
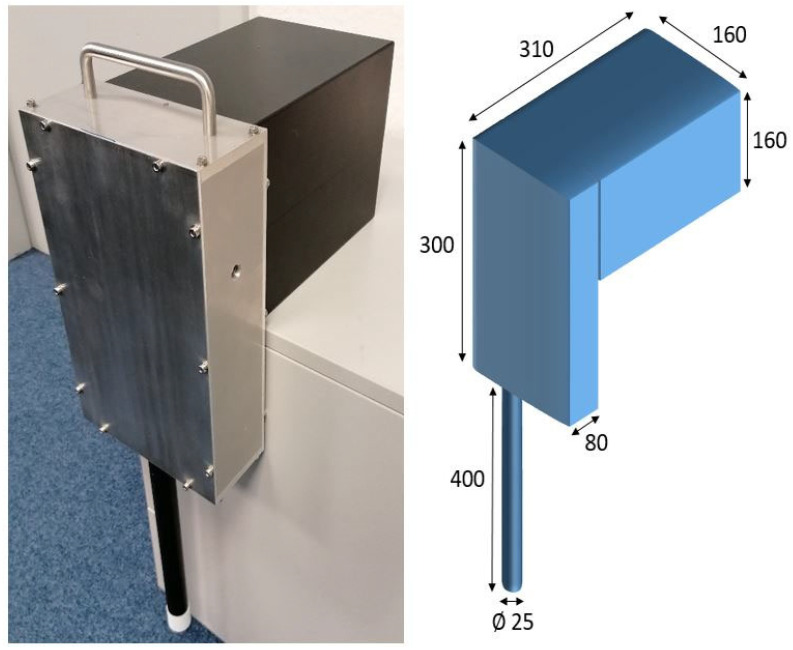
Image of the completed prototype in a custom aluminum housing. The bottom of the probe has a protective cap. All dimensions are given in mm.

The prototype was housed in an aluminum casing. The case was equipped with a carrying handle for portability. The total weight came to 8 kg. The housing and its dimensions can be seen in Figure 1.

**Figure 2:**
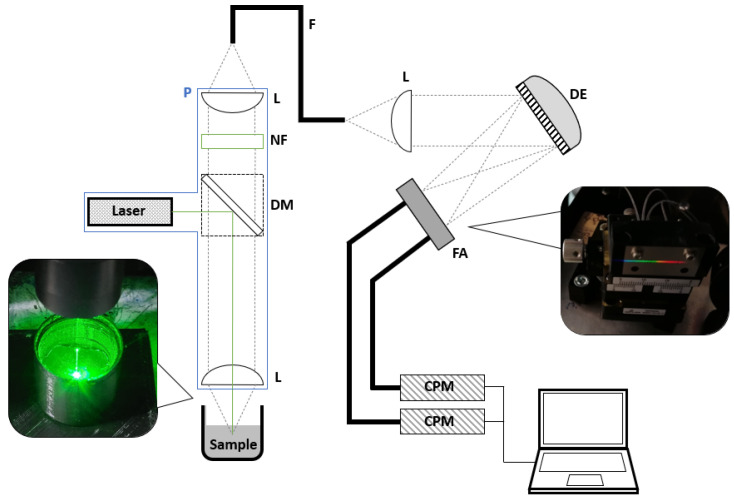
Schematic diagram of the Raman sensor prototype. P = probe, DM = dichroic mirror, NF = 532 nm notch filter, L = lens, F = fiber, DE = dispersive element, FA = fiber array, CPM = custom photon multiplier.

For the prototype presented, an excitation wavelength of 532 nm was specified. A spectrometer from Kaiser was used as the reference system (RXN1, Kaiser Optical Systems, Ann Arbor, MI, USA), which had the same emission wavelength and power output as the laser module used. A schematic representation of the measurement system is shown in Figure 2.

**Figure 3:**
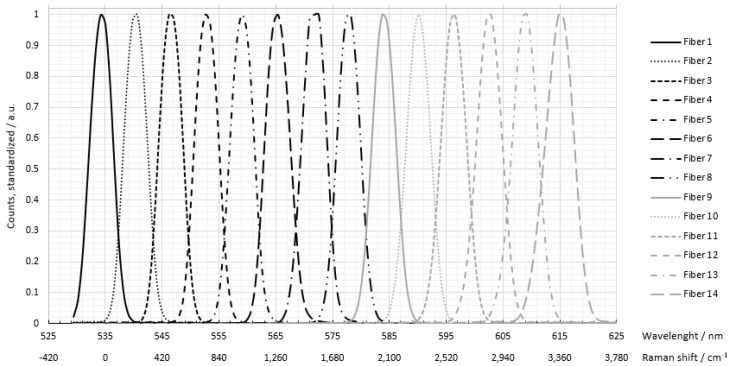
Resolution of the fiber array standardized and measured with a UV/VIS spectrometer (Zeiss, MCS621 VIS II, Jena, Germany). A halogen lamp (Zeiss, CLH600) was used as the light source.

The monochromatic light source was a collimated CW laser with an emission wavelength of 532 nm and an optical power output of 100 mW (CW532-100, Roithner Laser Technik, Wien, Austria). For potential application in explosive atmospheres, a 532 nm laser with 1 mW was also used (CW532-001, Roithner LaserTechnik). The laser was reflected into the confocal setup of the probe using a dichroic mirror (RT 532 rdc, AHF Analysetechnik, Tübingen, Germany). At the end of a 400 mm long probe head, the laser beam was focused by a planoconvex lens (f = 25 mm) into the sample. Photons generated by the Raman effect were scattered in all spatial directions. The Raman radiation generated in the lens focus was in turn collimated by the lens and guided back to the dichroic mirror. The Stokes shifted photons were able to pass the dichroic mirror due to their increased wavelength. A subsequent notch filter (zet532TopNotch, AHF Analysetechnik) blocked any remaining 532 nm photons. This was necessary because the laser had a much higher light output compared to the Raman effect, and therefore the filtering behavior of the dichroic mirror by itself was insufficient when considering reflexes and Rayleigh scattering. The filtered light was focused by a second planoconvex lens (f = 50 mm) onto an optical fiber (d = 600 µm). The focal length of the lens was chosen to be larger to fall below the numerical aperture of the optical fiber of NA = 0.42 (NA = 0.4 for lens) and to minimize light loss during coupling. In addition, this selection resulted in a doubling of the spot size. The optical fiber was mounted in a two-axis linear stage (CXY1Q, Thorlabs, Newtone, NJ, USA), which facilitated precisely the positioning of the fiber into the focus point of a second planoconvex lens to obtain the maximum signal. The optical fiber transferred the collected light to the photo spectrometric detection unit. There, the light transmitting from the optical fiber was first collimated with the previously mentioned third planoconvex lens (f = 25 mm) and projected onto a dispersive element. The dispersive element had a grating constant of 1300 lines per mm and mapped the resulting spectrum onto a flat plane. A linear fiber array consisting of 14 glass fibers (d = 600 µm) was positioned in this plane side by side in order to collect the dispersed Raman photons. The fiber mount was specifically designed for this setup. The fiber array was located on a rotary axis (RP01, Thorlabs) and on a two-axis linear stage (DSXYEG40, MiSUMi). This combination allowed the fiber array to be positioned precisely in-plane to optimize signal yield. Figure 3 shows the resolution of the individual optical fibers.

As displayed in Figure 3, the full width at half maximum of the fiber array was 5 nm or 150 cm^−1^. A slit aperture positioned directly in front of the fiber array could be used to further improve the resolution, as required by the substance peaks of interest, thus enabling selective detection. The width of the slits could be dimensioned as low as 50 µm with a height of 1 mm. However, the improved resolution would decrease signal intensity. The individual optical fibers of the fiber array were connected to three detection units. Due to the conditions of low light detection, custom photon multipliers (CPM) from Proxivision were selected [10]. The key features of CPMs are a low detection limit for light levels, a high dynamic range, and low background noise. A high gain enables the detection of even small numbers of photons. A further increase in sensitivity can be achieved by adjusting the cathode material for selective and large spectral ranges. Signal amplification by a factor of 108 places CPMs among the most sensitive photodetectors. The low noise of <10 counts per ms makes them particularly suitable for detecting low photon signals, as occur in the Raman effect. The high sensitivity of CPMs is at the same time a technical challenge, since stray light from other light sources, such as daylight, reflected light, or, in this application in particular, the excitation light of the laser also interferes with the measurement. It was therefore necessary to optically insulate the detection unit to near complete absence of light in the sensitive range. The resulting overcharge of the detector by primary electrons can lead to destruction of the dynode. To protect against this, an internal shutter was built in, which only allowed an exposure time of 180 ns. A dead time of 420 ns was used to neutralize the electrons. This automatic erasure and protection system determined the lowest integration time of CPMs at 1 ms specified by the manufacturer. To provide additional protection for the CPMs, the entire photo spectrometric detection unit was enclosed in a lightproof case with diffuse scattering black inner surfaces.

The operating software for the prototype was developed in LabView. The communication between individual components takes place via serial interface. The core functions of the software are the configuration of the measurement system and the evaluation of the measurement data. Through a graphical user interface, several results of the user specified measurement parameters can be displayed in real time. For the configuration of the measuring system there is a maintenance mode, which contains the core functions in reduced form and additionally a live view function, to assist with the calibration of the optical components. In the measuring mode, limit values of the batch processes of interest are stored as initial concentrations of the reactants and the target concentration of the product. If the limit values are reached during the process, an optical signal is sent to the operator. In addition, the temperature inside the prototype is monitored by temperature sensor. In the event of critical temperatures, a warning signal is issued first, followed by an emergency shut down of the measuring system if the pre-set limit value is exceeded. In this case, measurement data is backed up by an integrated emergency memory. The same applies in the event of a power failure.

For the experimental tests, several substance systems consisting of ammonium nitrate, di-sodium hydrogen phosphate, potassium sulfate, and ethanol were used, each dissolved in deionized water. The substances used are suitable reference systems, since they are used in industrial batch production of fertilizer and explosives as well as in food processing, among others [11,12,13].

**Figure 4:**
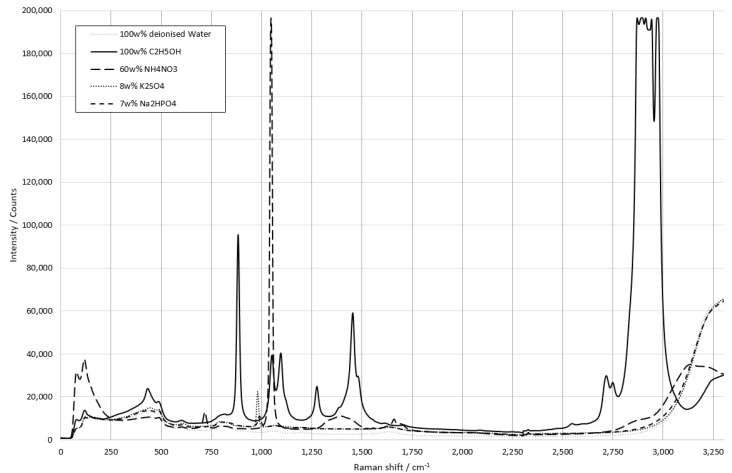
Raw spectra of ammonium nitrate, di-sodium hydrogen phosphate, potassium sulphate, and ethanol with the maximum concentration dissolved in deionized water. Integration time 10 s, spectra accumulation 3.

The pure substance spectra were recorded with a Raman spectrometer (RXN1, Kaiser Optical System, Ann Arbor, MI, USA) at a laser power of 100 mW and can be seen in Figure 4.

By measuring the individual raw materials and evaluating the measurement results, their Raman activity was confirmed, and the individual Raman peaks were identified. Table 1 matches the peaks to their associated vibration types. For the later evaluation only peaks below 1500 cm^−1^ were considered. This is due to the fact that in this setup the fingerprint range was prioritized for its higher information content on various covalent bonds. All of the Raman spectral data are shown in Figure 4 to illustrate all possible signal ranges of the setup, which was limited to a total range of 1600 cm^−1^. This range can be placed anywhere in the spectrum as chosen by the user.

All further measurements refer to the peaks listed here. All listed peaks agreed with the literature [14,15,16,17,18].

The substances used have different material properties, which are listed below in Table 2. The peak position was converted to nm from the determined values in Table 1. The remaining data were taken from the literature [19,20,21,22].

In the experimental tests, dilution series were produced, as listed in Table 2. Each series of measurements contained five predetermined concentrations limited by the maximum solubility and a set minimum of 1 w%. The difference of minimum and maximum was subdivided into five values. Thus, the linear behavior of the Raman effect in approx. equidistant steps was predicted based on the measurements. Table 3 shows the all concentration parameters of the individual substances.

## 3. Results

**Figure 5:**
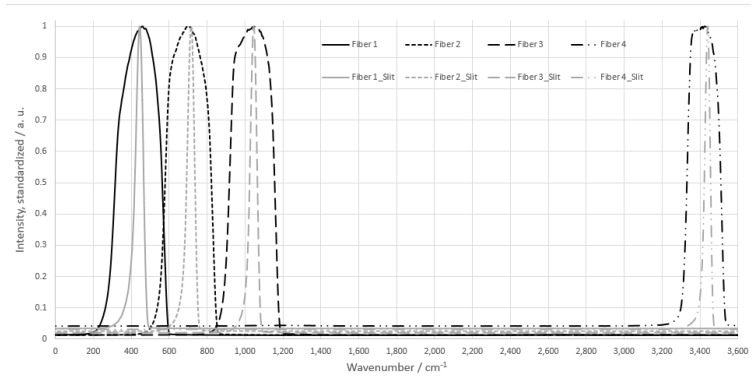
Reduction of the band width by slit mask in front of the fiber array standardized for visibility of improved spectral resolution. A halogen lamp was used as the light source, and the data were measured with a UV/VIS spectrometer (Zeiss, MCS621 VIS II, Jena, Germany).

First, the reduction of resolution by the slit aperture was investigated. The diameter of the glass fiber should have encompassed the entire height of the plane created by the dispersive and thereby reduce light loss. By superimposing the slit aperture onto the optical fibers, the light entering them was reduced, and the spectral resolution was increased. Figure 5 shows the latter effect.

As can be seen in Figure 5, the resolution of the individual optical fibers was reduced from 300 cm^−1^ to 50 cm^−1^. However, the overall signal intensity was reduced by a factor of 5 due to this artificial reduction of the fiber cross-section. An additional disadvantage was that only a single signal peak could be read out in the range of the bandwidth of individual fibers masked with the slit aperture. However, by appropriately positioning the edges of the slit aperture and the optical fibers, two closely spaced Raman peaks could be detected and thus increase the applicability. Due to the round shape of the optical fibers, the slits were located at the fiber edge when used in the described alignment, which inevitably led to a further loss of intensity. Accordingly, a trade-off between intensity and resolution was necessary for application-specific measurements.

**Figure 6:**
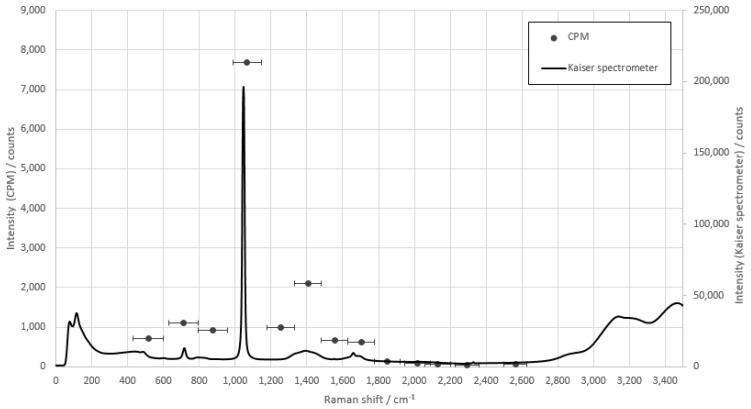
Comparison measurement of ammonium nitrate with the fiber array compared to the raw spectra of the Kaiser spectrometer. The integration time of the CPMs is 1 s, and the integration time of the Kaiser spectrometer is 10 s.

First, the fiber array had to be adjusted to the individual substances and the maximum signal intensity determined. For this purpose, the measurements of the individual substances with the prototype were compared with the measurements of the raw spectra. This was to approximate the spectrum of the substances with the CPMs and to select the correct glass fiber for the individual peaks. Figure 6 shows this for ammonium nitrate. The single points show the bandwidth of the optical fiber and which range is detected by the connected CPMs, since the detectors registered and counted all incoming photons within their sensitivity range, regardless of the respective wavelength. 

The measurements of ammonium nitrate show that glass fiber 4 in the array registered the specific peak at 1047 cm^−1^ and was therefore used for the concentration measurements. As with all other measurements, the individual substances had significant peaks in the fingerprint range up to 1500 cm^−1^ and will be used for further consideration.

**Figure 7:**
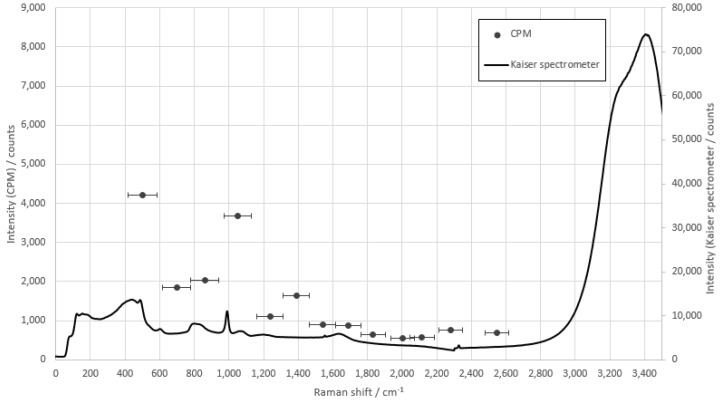
Comparison measurement of di-sodium hydrogen phosphate with the fiber array compared to the raw spectra of the Kaiser spectrometer. The integration time of the CPMS is 1 s, and the integration time of the Kaiser spectrometer is 10 s.

The measurement of di-sodium hydrogen phosphate gave the result shown in Figure 7.

In contrast to ammonium nitrate, the measurement of sodium phosphate showed the highest value for glass fiber 1. However, the peak in this range was not suitable due to the presence of a broadband background and a varying slope. Glass fiber 4, however, showed a comparable signal intensity for the phosphate specific peak at 995 cm^−1^ and was selected for concentration measurements.

**Figure 8:**
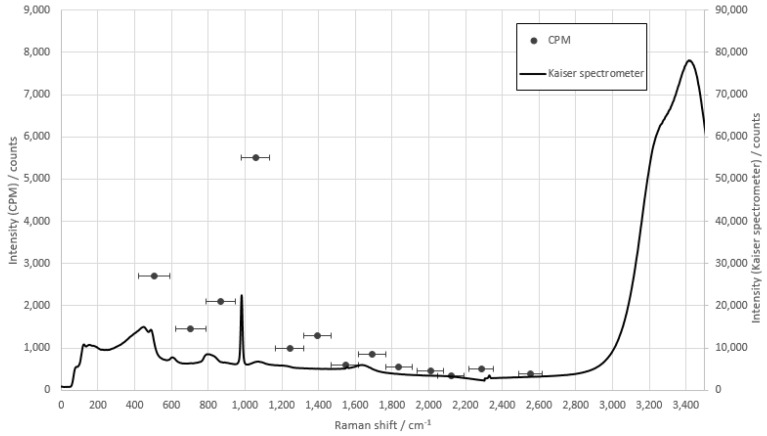
Comparison measurement of potassium sulphate with the fiber array compared to the raw spectra of the Kaiser spectrometer. The integration time of the CPMs is 1 s, and the integration time of the Kaiser spectrometer is 10 s.

The measurement of potassium sulfate using the described method is shown in Figure 8. The significant peak at 982 cm^−1^ of potassium sulfate coincided with glass fiber 4 and served as the basis of the concentration series.

**Figure 9:**
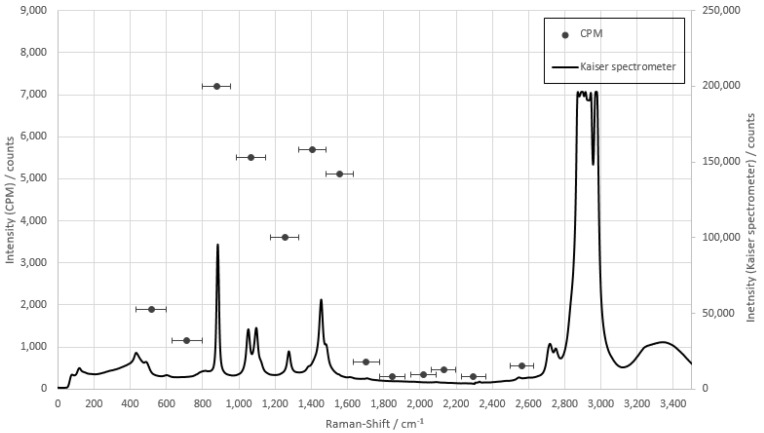
Comparison measurement of ethanol with the fiber array compared to the raw spectra of the Kaiser spectrometer. The integration time of the CPMS is 1 s, and the integration time of the Kaiser spectrometer is 10 s.

For ethanol, there were several peaks that could be selected for further measurements (Figure 9). The most obvious peak was at 882 cm^−1^, which was detected by glass fiber 3. Alternatively, optical fiber 6, and thus the peak at 1454 cm^−1^ was eligible. In this measurement, several peaks from the CH sum peak could be seen in the range from 2800 to 3000 cm^−1^, showing the strongest signal. However, this peak was unspecific and out of range in the current configuration of the set up.

Table 4 lists the assignment of optical fibers to specific substance peaks. This served as a basis for the design of the measurement system and demonstrated that individual optical fibers could be assigned to individual substance signals.

**Figure 10:**
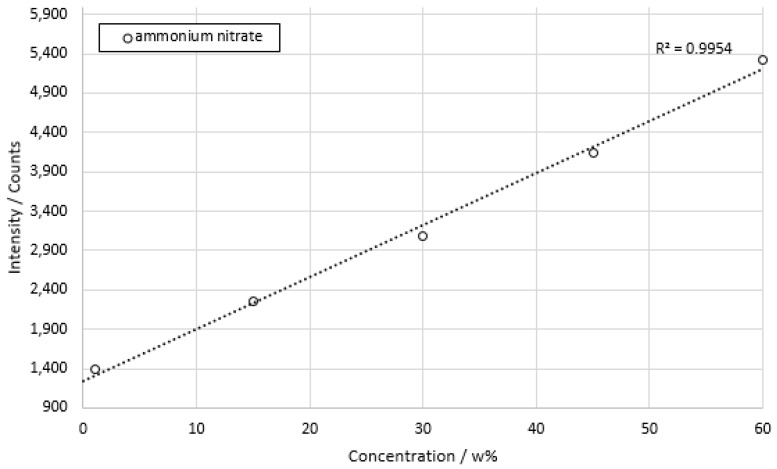
Concentration series of ammonium nitrate dissolved in deionized water. Integration time 100 ms; total measuring time 2 min per concentration.

The concentration series measurements were carried out based on Table 4. Measurement data were acquired with an integration time of 100 ms over a period of 2 min for all samples. The laser was set to a power output of 100 mW. Five measurement series were recorded per concentration. The measurement results were then averaged, and the mean values are shown in the following figures starting with ammonium nitrate in Figure 10.

The measurement data of ammonium nitrate achieved a coefficient of determination of *R*^2^ = 0.9954. The calculated limit value of the minimum concentration of ammonium nitrate for a linear course was 1.01 w% or 130 mmol/L at a resolution of 23 counts. The standard deviation for the measurement shown ranged from 2.72% at the minimum concentration to 1.52% at the maximum concentration.

**Figure 11:**
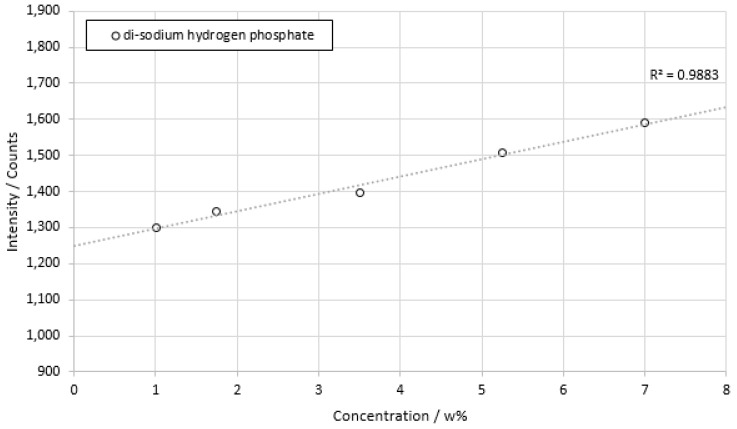
Concentration series of di-sodium hydrogen phosphate dissolved in deionized water. The integration time is 100 ms. Each concentration was measured for about a time of 2 min.

The next concentration series showed the results of sodium phosphate dilution (Figure 11). 

The coefficient of determination for sodium phosphate measurements was calculated as *R*^2^ = 0.9883, which also indicated an approx. linear behavior. The standard deviation for the measurement shown ranged from 1.47% of the average signal at 3.5 w% to 3.53% of the average signal at 1.75 w%. The minimum resolution, based on the concentration difference of the concentration series, was 0.75 w%, which corresponded to a resolution of 50 mmol/L. 

**Figure 12:**
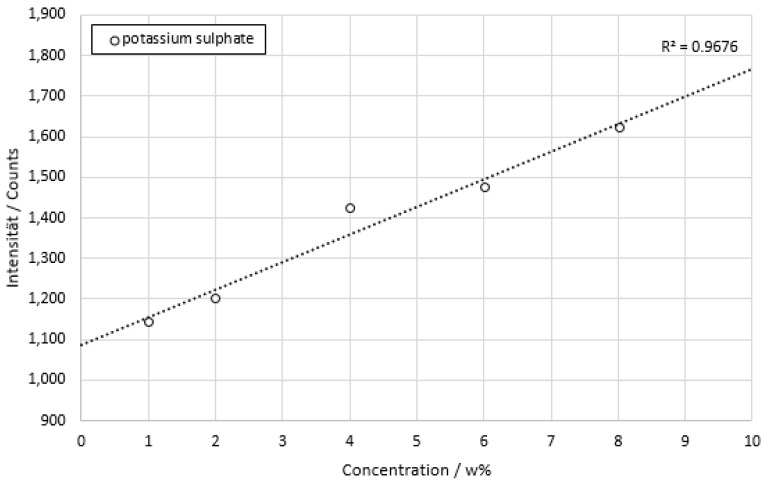
Concentration series of potassium sulphate dissolved in deionized water. The integration time is 100 ms. Each concentration was measured for about a time of 2 min.

The data of potassium sulphate measurements are shown in Figure 12.

The plotted concentration curve of potassium sulfate showed a linear regression with *R*^2^ = 0.9676. The standard deviation ranged from 2.39% of the average signal at 2 w% to 3.73% of the average signal at 1 w%. The minimum resolution between concentration levels was 1 w% or 60 mmol/L, respectively.

**Figure 13:**
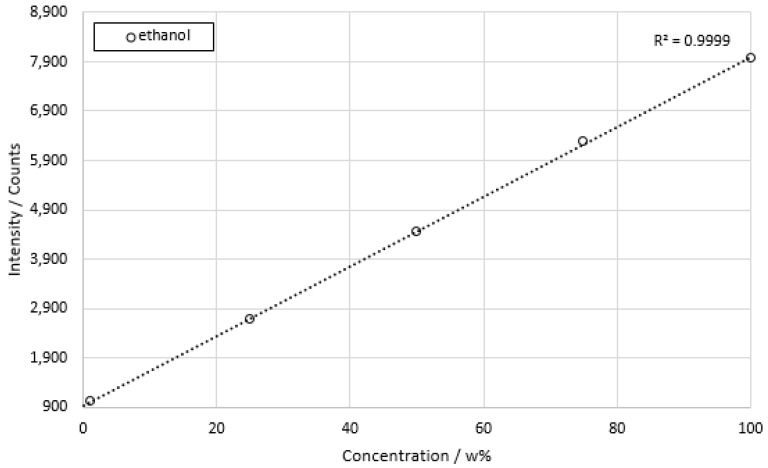
Concentration series of ethanol in deionized water. Integration time 100 ms. Total measuring time per concentration approx. 2 min.

Finally, the measurement of ethanol dissolved in deionized water is shown in Figure 13. The highest Pearson coefficient off all investigated dilution series was achieved in this experimental set.

A unique feature of ethanol in comparison to the other substances was the accessible concentration range from 100% to 1%, with ethanol decrements of 25% per dilution. This resulted in a coefficient of determination of *R*^2^ = 0.9999. Ethanol thus showed the highest agreement with linear behavior of all the substances measured. The standard deviations were between a minimum of 4.18% at a concentration of 1 w% and a maximum of 9.82% at a concentration of 50 w%. The minimum resolution was 1 w%. This corresponded to 210 mmol/L.

In an additional series of measurements, concentration series of ammonium nitrate and potassium sulfate were measured with a 1 mW laser to show the eligibility of the prototype for application in explosive atmospheres. The use of highly sensitive CPMs enabled Raman photons to be detected even at very low excitation power conditions. At the same time, the required effort to install the presented prototype was estimated to be low compared to conventional EX-range measuring equipment.

**Figure 14:**
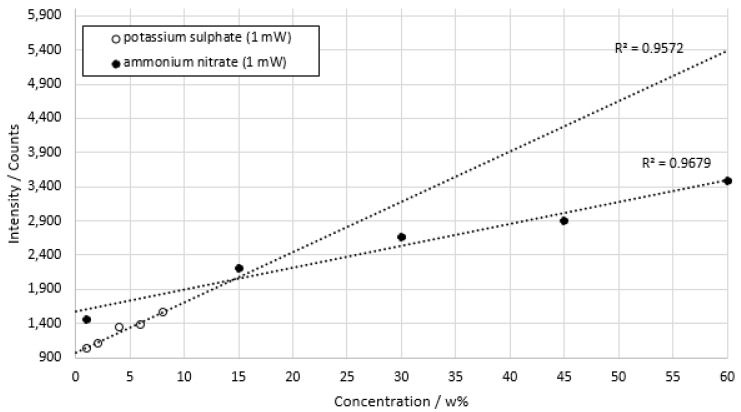
Concentration series of ammonium nitrate and potassium sulphate in deionized water. Integration time 6000 ms. Measuring time per concentration approx. 2 min. Laser power 1 mW.

Five measurements were performed per concentration with a duration of 2 min per measurement. The integration time was 6 s. Figure 14 shows the results of low power measurements of ammonium nitrate and potassium sulfate.

At 1 mW excitation power, the coefficient of determination for ammonium nitrate was *R*^2^ = 0.9679. The coefficient of determination for potassium sulfate was *R*^2^ = 0.9572. An approximately linear relationship of signal intensity and concentration was shown for both substances. Again, concentration differences of up to 50 mmol/L could be measured. The standard deviations of potassium sulphate were between a minimum of 2.85% at a concentration of 6 w% and a maximum of 4.16% at a concentration of 1 w%. For ammonium nitrate, the standard deviation had a minimum of 0.74% at a concentration of 15 w% and a maximum of 2.87% at a concentration of 30 w%.

In the following section, the results of the measurements are summarized in a comprehensive overview.

## 4. Discussion

The presented portable, high-resolution, photo spectrometric Raman prototype for monitoring of batch processes was validated with dilution series of the substances ammonium nitrate, sodium phosphate, potassium sulfate, and ethanol, each dissolved in deionized water. Concentration levels could be detected for all substances down to a minimum resolution of 55 mmol/L. Low integration times were realized by using highly sensitive CPMs. Additionally, the use of a slit aperture in combination with a fiber array resulted in an increased spectral resolution at the cost of reduced signal intensity. In general, this combination resulted in two optional set-up variants that are cost-effective and easy to implement. The first variant consisting only of the fiber array has a low resolving power at high intensities, which is useful for broad Raman peaks or measuring ranges. The second variant adds a slit aperture resulting in a high resolving power at low signal intensities. Both parameters can be varied by the slit width. Due to the flexible design, various reactions can be tracked. The slit aperture is an inexpensive alternative to ensure versatility.

The measurements of the individual substance systems showed that there is very good linearity in the concentration measurements with coefficients of determination ranging from *R*^2^ = 0.9676 for potassium sulphate to *R*^2^ = 0.9999 for ethanol. Therefore, concentration differences as low as 0.75 w% can be detected for di-sodium hydrogen phosphate. Further investigations are needed to improve the minimum concentration resolution. 

## 5. Conclusions

This article focuses on the functional capabilities of the presented measurement system and its versatility in batch process applications. Due to the measurement with a laser with 1 mW optical power, the presented system can also be applied in hazardous areas. Likewise, the system can be used in continuous processes due to low integration times and high sensitivities. Further investigations for more detailed statements will follow in a later study.

The next steps include the adaptation of the prototype to future requirements, an optimization of the spectral resolution to be able to better differentiate substances with closely spaced peaks, as well as an optimization of the constructive elements regarding broader applicability.

## Figures and Tables

**Table 1:** Raman band assignment of the individual substances below a Raman shift of 1500 cm^−1^.

Ammonium Nitrate (cm^−1^)	Type of Vibration	Di-Sodium Hydrogen Phosphate (cm^−1^)	Type of Vibration	Potassium Sulphate (cm^−1^)	Type of Vibration	Ethanol (cm^−1^)	Type of Vibration
715	$\nu_{4}\left(NO_{3}^{-}\right)$	995	$\nu_{1}\left(HPO_{4}^{2-}\right)$	449	$\nu_{2}\left(SO_{4}^{2-}\right)$	882	$\nu_{1}\left(C-C\right)$
1047	$\nu_{1}\left(NO_{3}^{-}\right)$			616	$\nu_{4}\left(SO_{4}^{2-}\right)$	1054	$\nu_{1}\left(C-O\right)$
1288	$\nu_{3}\left(NO_{3}^{-}\right)$			982	$\nu_{1}\left(SO_{4}^{2-}\right)$	1099	$\nu_{2}\left(-CH_{3}\right)$
1416	$\nu_{3}\left(NO_{3}^{-}\right)$			1107	$\nu_{3}\left(SO_{4}^{2-}\right)$	1277	$\nu_{3}\left(-CH_{2}\right)$
1459	$\nu_{4}^{\prime }\left(NH_{4}^{+}\right)$			1145	$\nu_{3}\left(SO_{4}^{2-}\right)$	1454	$\nu_{2}\left(-CH_{3}\right)$

**Table 2:** Substance specifications of ammonium nitrate, di-sodium hydrogen phosphate, potassium sulphate, and ethanol.

	Ammonium Nitrate	Di-Sodium Hydrogen Phosphate	Potassium Sulphate	Ethanol	Water
Molecular formula	NH_4_NO_3_	Na_2_HPO_4_	K_2_SO_4_	C_2_H_5_OH	H_2_O
Peak position (nm)	563.38	561.26	561.26	557.97	650.75
Density $(\frac{g}{l}$)	1720	1700	2660	790	998
Water solubility ($\frac{g}{l}$)	1920	77	110	-	-
Solubility limit (w%)	65.80	7.16	9.93	-	-

**Table 3:** Used concentrations of the individual substances dissolved in deionized water.

NH_4_NO_3_/w%	Na_2_HPO_4_/w%	K_2_SO_4_/w%	C_2_H_5_OH/w%
60	7	8	100
45	5.25	6	75
30	3.5	4	50
15	1.75	2	25
1	1	1	1

**Table 4:** Selection of glass fibers related to the selected peak in the substances to be investigated.

Substance	Fiber Position	Raman Peak (cm^−1^)
NH_4_NO_3_	4	1047
Na_2_HPO_4_	4	995
K_2_SO_4_	4	982
C_2_H_5_OH	3	882

## Data Availability

Not applicable.
